# Memory B-Cell Responses Against Merozoite Antigens After Acute *Plasmodium falciparum* Malaria, Assessed Over One Year Using a Novel Multiplexed FluoroSpot Assay

**DOI:** 10.3389/fimmu.2020.619398

**Published:** 2021-02-12

**Authors:** Peter Jahnmatz, Christopher Sundling, Victor Yman, Linnea Widman, Muhammad Asghar, Klara Sondén, Christine Stenström, Christian Smedman, Francis Ndungu, Niklas Ahlborg, Anna Färnert

**Affiliations:** ^1^ Division of Infectious Diseases, Department of Medicine Solna, Karolinska Institutet and Center for Molecular Medicine, Stockholm, Sweden; ^2^ Mabtech AB, Nacka Strand, Sweden; ^3^ Department of Infectious Diseases, Karolinska University Hospital, Stockholm, Sweden; ^4^ Division of Biostatistics, Institute of Environmental Medicine, Karolinska Institutet, Stockholm, Sweden; ^5^ Department of Clinical Microbiology, Karolinska University Hospital, Stockholm, Sweden; ^6^ Kenya Medical Research Institute (KEMRI)/Wellcome Trust Research Programme, Kilifi, Kenya; ^7^ Department of Molecular Biosciences, The Wenner-Gren Institute, Stockholm University, Stockholm, Sweden

**Keywords:** antibody, memory B-cell, FluoroSpot, *P. falciparum* malaria, recombinant proteins

## Abstract

Memory B cells (MBCs) are believed to be important for the maintenance of immunity to malaria, and these cells need to be explored in the context of different parasite antigens and their breadth and kinetics after natural infections. However, frequencies of antigen-specific MBCs are low in peripheral blood, limiting the number of antigens that can be studied, especially when small blood volumes are available. Here, we developed a multiplexed reversed B-cell FluoroSpot assay capable of simultaneously detecting MBCs specific for the four *Plasmodium falciparum* blood-stage antigens, MSP-1_19_, MSP-2, MSP-3 and AMA-1. We used the assay to study the kinetics of the MBC response after an acute episode of malaria and up to one year following treatment in travelers returning to Sweden from sub-Saharan Africa. We show that the FluoroSpot assay can detect MBCs to all four merozoite antigens in the same well, and that the breadth and kinetics varied between individuals. We further found that individuals experiencing a primary infection could mount and maintain parasite-specific MBCs to a similar extent as previously exposed adults, already after a single infection. We conclude that the multiplexed B-cell FluoroSpot is a powerful tool for assessing antigen-specific MBC responses to several antigens simultaneously, and that the kinetics of MBC responses against merozoite surface antigens differ over the course of one year. These findings contribute to the understanding of acquisition and maintenance of immune responses to malaria.

## Introduction

Acquisition of clinical immunity to malaria is achieved after multiple infections in individuals living in areas with high transmission (1, 2). The protective role of antibodies to blood stage infections was demonstrated through passive transfer studies (3). However, due to the large number of proteins expressed during the parasite life cycle, it is important to understand to what antigens antibodies need to be directed in order to establish protection. Moreover, maintaining protection without repeated exposure is however challenging as antibody levels against parasite antigens wane rapidly after both natural infections and vaccination (4–6).

Memory B cells (MBCs) are considered long-lived and can be maintained for decades in the absence of circulating antibodies (7). At a healthy state, MBCs are quiescent while circulating through the vasculature and secondary lymphoid organs. Upon pathogen re-exposure, these MBCs rapidly proliferate and differentiate into plasma blasts that produce affinity matured antibodies against the pathogen (8–10). In the context of malaria, antigen-specific MBCs have been detected in peripheral blood using the widely established ELISpot assay (11) and have been identified as marker of past exposure (12, 13). Nevertheless, the relative importance of MBCs in protective immunity to *P. falciparum* as well as the kinetics and breadth of MBC responses after a natural infection is yet to be elucidated. Few studies have investigated MBCs longitudinally within an individual after a malaria episode (14, 15). Nonetheless, studies of antigen specific memory in malaria endemic areas are challenged by repeated infections. By studying MBC responses in travelers returning with malaria to a country without transmission, the immune response after a natural infection can be assessed in the absence of re-exposure. We have previously shown, using a cross-sectional approach, that after a single infection, MBCs specific for *P. falciparum* blood stage antigens can be maintained by some individuals for many years without re-exposure (11). In order to further investigate how antigen-specific immune responses are acquired and maintained after acute malaria, we set up a prospective longitudinal cohort study of travelers sampled several times over one year after treatment.

Considering the low frequencies of antigen-specific MBCs in circulation, and the need to study multiple *P. falciparum* antigens as well as limited sample volumes, especially in children, novel highly sensitive tools are required for analysis. Recently, we described a reversed B-cell FluoroSpot assay for simultaneous detection of human antigen-specific MBCs against three different pathogens (16). Here, we adapted this multiplex reversed B-cell FluoroSpot assay for simultaneous detection of MBCs specific to four *P. falciparum* merozoite antigens; MSP1_19_, MSP-2, MSP-3 and AMA-1. These antigens have been extensively studied as potential candidate antigens for a blood stage vaccine, as well as markers of exposure in seroprevalence studies (17–19). We used the assay to study the kinetics of antigen-specific MBC responses in travelers treated for natural *P. falciparum* malaria and followed over one year in a malaria-free setting. We also explored the capacity of a newly developed FluoroSpot reader to estimate the individual spot intensity and volume as a new dimension of MBC responses to individual antigens.

## Materials and Methods

### Study Population

**Figure 1:**
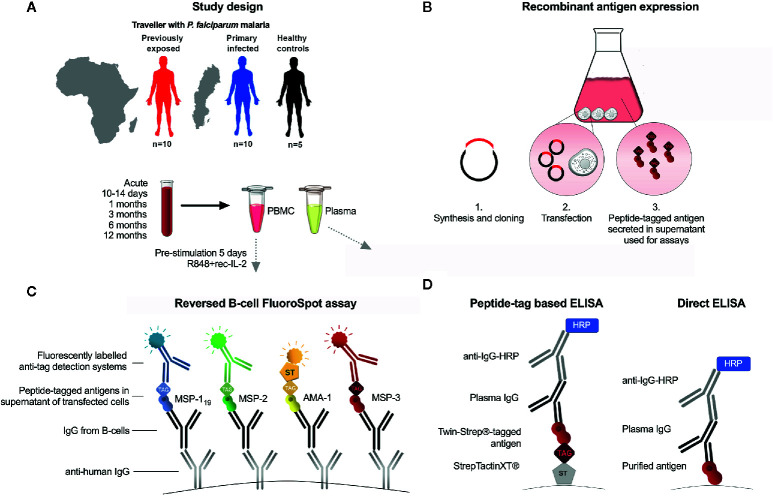
Schematic presentation of study and experimental setup. **(A)** Study participants were adult patients with acute *Plasmodium falciparum* malaria (previously exposed, n=10, first time infected n=10) and non-exposed controls (n=5) and 5 non-exposed controls. Peripheral blood mononuclear cells (PBMC) and plasma were isolated at time of diagnosis (acute), and at 10 days, 1, 3, 6 and 12-months follow-up after treatment. **(B)** Expression of recombinant peptide-tagged antigens and supernatant added to the FluoroSpot assay. **(C)** Principles of reversed B-cell FluoroSpot assay. Immobilized anti-human IgG antibodies coated to wells were used to capture antibodies secreted by B cells. B cell derived antibodies were subsequently allowed to bind antigens tagged with different peptides in order to determine the antigen-specificity of the B cell. The position of a B-cell with a given specificity was determined using fluorescently labeled peptide-specific detection systems. **(D)** Peptide tag-based ELISA and direct ELISA.

Adults presenting with fever and other symptoms after visiting tropical areas and diagnosed with *P. falciparum* malaria at Karolinska University Hospital in Stockholm, Sweden were invited to participate in a prospective study (5, 20) (**Supplementary Table 1**). Patients were enrolled at diagnosis (acute time point) and asked to return for follow-up sampling on 10 days, 1, 3, 6 and 12 months after treatment (**Figure 1A**). Data regarding previous malaria infections as well as medical and travel history were collected using a questionnaire. For the purpose of this study, we selected ten individuals reporting their first episode of malaria (referred to as “primary infected”), and ten individuals with repeated previous exposure (referred to as “previously exposed”). These individuals were selected based on having samples from at least four follow-up samples and reporting no travel to areas with malaria transmission during the one year follow up period. All patients were treated with a full course of artemether–lumefantrine (Riamet^®^, Novartis). Patients who suffered from treatment failure and presented with recrudescent malaria during treatment, were successfully treated with a second course of either artemether–lumefantrine (Riamet^®^) or mefloquine (Lariam^®^, Roche). The primary infected individuals were all Swedish natives with no previous malaria diagnosis, and no or only short-term cumulative residency in malaria endemic areas (range 0-2 years). The previously exposed individuals were Swedish residents born in Sub-Saharan Africa who previously lived in a malaria-endemic area for an extended period of time (range 18-39 years) and who reported that they had multiple malaria infections before moving to Sweden. Also, five healthy adults who had never visited malaria endemic areas were included as unexposed controls. In addition, 1-month follow-up samples from five adults of African origin within the cohort with high plasma levels of antibodies against the four selected antigens (5), were selected for optimization of the B-cell FluoroSpot assay.

### Preparation of Samples

Venous blood (40 mL) was collected in sodium-heparin or EDTA tubes (BD, Franklin Lakes, NJ, USA) at each visit. Peripheral blood mononuclear cells (PBMC) were isolated from 35 mL whole blood using Ficoll density centrifugation as previously described (20). Five millilitres of blood were used for preparation of plasma. Isolated PBMC and plasma samples were stored at −150°C and −80°C, respectively. All work on living cells was performed in laminar hoods in a BSL2 protection lab.

### Antigen Preparation

*P. falciparum* merozoite antigens were expressed recombinantly in a mammalian expression system as previously described (21), before use in the FluoroSpot assay as well as ELISA (**Figure 1B**). Antigens were expressed based on the following protein sequences: MSP-1_19_, Uniprot accession number Q8I0U8; MSP-2 (3D7-type), P50498; MSP-2 (FC27-type), P19599; MSP-3, Q8IJ55 and AMA-1, Q7KQK5. For MSP-2, the 3D7 and FC27 type antigens were expressed and tagged with the same peptide tag and referred to as MSP-2 when used in combination at equal amounts. The antigens were tagged with the following peptide tags described previously (22): MSP-1_19_ with BAM; MSP-2 (FC27 and 3D7), with GAL; MSP-3 with WASP and AMA-1 with TWIN-Strep-tag^®^ (IBA LifeSciences, Goettingen, Germany) (amino acid sequence WSHPQFEKGGGSGGGSGGSAWSHPQFEK). In potential N-linked glycosylation sites, (NXS/T), serine (S) or tyrosine (T) were replaced by alanine (A) to avoid epitope interference. The mouse IgG kappa leader sequence (METDTLLLWVLLLWVPGSTGD) was added to each sequence to ensure protein secretion. Also, tag control antigens; cow IFN-γ tagged with peptide tag BAM, woodchuck IFN-γ tagged with GAL, dog IFN-γ tagged with WASP, and horse IFN-γ tagged with TWIN-Strep-tag^®^ were expressed separately as described (22). Each *P. falciparum* antigen was also separately expressed with a TWIN-Strep-tag^®^ (IBA LifeScience) at the N-terminus to be used for ELISA. Synthesis and cloning of sequences into plasmid pcDNA3.1/Zeo(−) (Thermo Fisher Scientific, Waltham, MA, USA Technologies) was performed by GeneScript (Piscataway, NJ, USA). Transient transfection was performed using the Expi293 transfection system (Thermo Fisher Scientific) as previously described (23). After 6 days, cell supernatants were harvested and stored in −20°C until use.

### Reversed B-Cell FluoroSpot Assay

A schematic presentation of the FluroSpot assay is shown in **Figure 1C**. Low fluorescent 96-well polyvinylidene fluoride plates (Millipore, Bedford, MA, USA) were prewetted with 20 μL/well 35% ethanol for one minute then washed with 200 μL/well H2O. Next, 100 μL mouse anti-human IgG specific for the Fc region (mAb MT91/145 Mabtech) diluted to 15 μg/mL in sterile PBS was added to wells followed by incubation, washing and blocking as previously described (22). The culture medium used for blocking was then replaced by 100 μL/well of fresh medium containing 250,000 cells that had previously been cultured for 5 days at 37°C and 5% CO2 in the presence of 1 μg/mL R848 and 10 ng/ml of recombinant interleukin (IL) 2 (both from Mabtech). The plates were incubated for 24 h at 37°C with 5% CO2. After the plates had been washed, 100 μL of peptide-tagged parasite antigens diluted in 0.1% BSA (PBS/BSA) were added to the wells and incubated for 1 hr at RT. As a control for possible background binding to the tag, supernatants from irrelevant antigens (horse IFN-γ, woodchuck IFN-γ, dog IFN-γ, and cow IFN-γ) tagged with the same peptide tags as the parasite antigens, were added to separate wells. Optimal concentrations of each antigen were determined by testing serial dilutions of the respective antigen in the FluoroSpot using cells from study subjects with known high-level IgG antibody responses against the selected antigens (5). Plates were incubated for 1 hr and then washed as described above. Next, 100 μL of fluorophore-conjugated antibodies, anti-BAM-405, anti-GAL-490 and anti-WASP-640 (all from Mabtech AB), as well as fluorophore-conjugated Strep-Tactin^®^XT (IBA LifeSciences) (STXT-550), all diluted to 0.5 µg/mL in PBS/BSA supplemented with filtered human plasma diluted 1:100, were added to the wells and incubated for 1 hr at RT. Human plasma was added to block coating antibody from potentially binding anti-tag detection mAb. The frequency of total IgG-producing cells was analysed using 25,000 pre-stimulated PBMCs in two separate duplicate wells. To detect IgG-producing cells, 100 μL/well of biotinylated mAb anti-human IgG (MT78/145-biotin) diluted to 0.5 μg/mL, were added followed by Streptavidin-550 in PBS/BSA. After a final wash, 50 μL of Fluorescent enhancer (Mabtech AB) was added to plates and incubated for 10 minutes at RT before the plates’ underdrain were removed and plates were left to dry in the dark at RT. The FluoroSpot plates were analysed using the Mabtech IRIS™ reader system equipped with Apex™ software version 1.1.7. The relative spot volume (RSV) of each counted spot was determined by the Apex™ and presented as arbitrary units. Definition of assay disqualification of a sample was set to <1000 total IgG spot forming units (SFU) per 250,000 cells. The threshold for MBC positivity was defined as the mean reactivity of healthy unexposed controls plus 2 standard deviations (SD). The percentage of antigen-specific MBCs was calculated by dividing the number of antigen-specific spots with the number of total IgG spots.

### Peptide Tag-Based ELISA

Antibody levels, in plasma samples collected in parallel with the PBMCs at each time point, against antigens MSP-1_19_, MSP-2 (3D7), MSP-2 (FC27), MSP-3, AMA-1, as well as a tagged control antigen (horse IFN-γ), were analysed by ELISA. The antigens were expressed with the same amino acid sequence as the antigens used for FluoroSpot with the difference that they were all tagged with a TWIN-Strep^®^ tag. High-binding polystyrene 96-well ELISA plates were coated with 100 μL/well of Strep-Tactin^®^XT (IBA BioScience) diluted to 1 μg/mL in PBS and incubated overnight. The following day, plates were blocked with 100 µL incubation buffer consisting of PBS supplemented with 0.1% BSA and 0.1% Tween 20 (both from Sigma-Aldrich, Saint Louis, MO, USA), for 1 hr. Next, the plates were washed using an automated ELISA washer, followed by the addition of 100 μL TWIN-Strep^®^ tagged variants of the above-mentioned antigens diluted 1:5 in incubation buffer. Next, the plates were washed using an automated ELISA washer, followed by the incubation of 100 µL plasma diluted to 1:2000 in incubation buffer for 1hr at RT. Following wash, 100 µL of horseradish peroxidase conjugated mouse anti-human IgG mAb (Mabtech AB) diluted to 0.5 µg/mL in incubation buffer was added to the plates and incubated for 1 hr and then washed as above. Development of signal was done by adding 100 µL 3,3’,5,5’-Tetramethylbenzidine (TMB) substrate solution (Mabtech AB) to the plates for 10 min, after which the reaction was stopped by addition of 100 µL ELISA Stop solution (Mabtech AB). Plates were read in an Emax ELISA reader at 450 nm and analysed using ELISA software SoftMax pro (Molecular Devices, San Jose, CA, USA). The threshold for seropositivity was defined as the mean reactivity of five healthy malaria unexposed controls plus 2 SD.

In order to compare the antigenicity of the expressed antigens with peptide tags and purified antigens, an indirect ELISA was performed using the purified *E.coli*-derived antigens described elsewhere (24). In brief, ELISA plates were coated with purified *E.coli*-derived MSP-1_19_, MSP-2_CH150/9 (corresponding to the 3D7 allele), MSP-2_Dd2 (corresponding to the FC27 allele), MSP-3_3D7 or AMA-1_3D7 at 1 μg/mL in PBS and incubated overnight. After blocking and washing, a pool of plasma from individuals considered semi-immune against *P. falciparum* malaria was added in serial dilutions. The plates were developed and analysed as described for the peptide tag-based ELISA. A schematic presentation of the two ELISA formats is shown in **Figure 1D**.

### Statistical Analyses

Statistical analyses were performed using GraphPad Prism version 8.3 (GraphPad Software, La Jolla, CA) and STATA MP version 16.0. The Mann-Whitney U-test was used to compare reproducibility between tests, duplicates and singleplex vs multiplex regarding SFU and RSV as well as differences in %MBC/total IgG or antibody levels between categorical timepoints. Spearman correlation was used to analyse associations between logarithmically transformed %MBC/total IgG, RSV or antibody levels throughout the study period, associations between immunogenicity of newly expressed antigens and *E. coli*-derived purified antigens, associations between %MBC/total IgG and SFU (goodness of fit was shown as r), and variability between replicate wells. A mixed-effects linear regression model adjusted for categorical time point of sample together with subject specific random intercept and time slope was used to compare differences between groups regarding %MBC/total IgG, RSV, antibody levels, SFU total IgG, and breadth, but also if MBC responses in patients suffering from treatment failure differed from other patients in that group. A p value <0.05 was considered as significant.

## Results

### MBC Responses to *P. falciparum* Merozoite Antigens

Four *P. falciparum* merozoite antigens were expressed recombinantly in a mammalian expression system with either a peptide or TWIN-Strep^®^ tag. Antigen expression was verified by Western Blot, with bands detected at expected molecular weights (**Supplementary Figure 1A**). The antigenicity of the expressed antigens, using *P. falciparum*-reactive plasma in peptide tag ELISA, was highly comparable to purified *E. coli*-derived antigens in an indirect ELISA (**Supplementary Figure 1B**).

**Figure 2:**
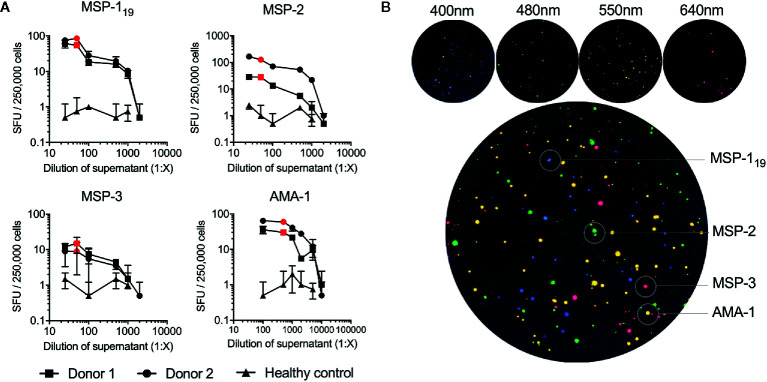
Titration and optimization of expressed antigens used in the FluoroSpot assay. **(A)** The optimal dilution of each antigen was determined by titration in the B-cell FluoroSpot assay. The optimal dilution of an antigen was defined as the dilution where the number of spot-forming units (SFU) obtained plateau levels without increasing background noise. Each antigen was tested with PBMC from two separate control donors with high antibody responses and a healthy control. The optimal concentration of the antigen used in the final FluoroSpot assay is displayed as a red dot in the graph (MSP-1_19_ 1:50 dilution of supernatant, MSP-2 1:50 3D7 combined with 1:50 FC27, MSP-3 1:50, and AMA-1 1:500). **(B)** Individual filter images and emission wavelength as wells as computerized overlay of images from a single B-cell FluoroSpot readout with a donor responding to all parasite antigens. Blue spots represent spot-forming units (SFU) from memory B cells specific against MSP-1_19_, green; MSP-2, red; MSP-3 and yellow; AMA-1.

**Figure 3:**
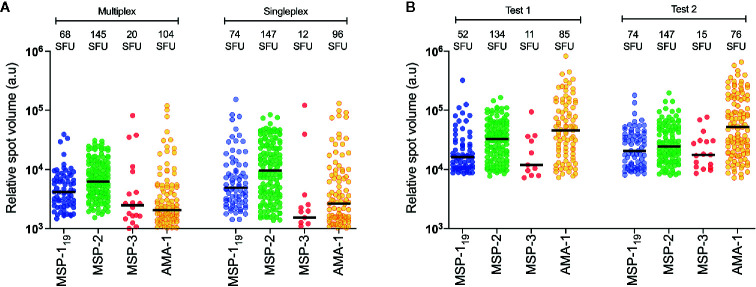
Reproducibility between test. **(A)** Comparison of frequency of spot-forming units (SFU) and relative spot volume (RSV) of individuals spots counted against different antigens in a single individual (patient id 2014009) with antigens tested together (multiplex) or in separate wells (singleplex). **(B)** Comparison of frequency of SFU and RSV of individuals spots counted against different antigens at two separated test occasions using B-cell FluoroSpot with one single individual (patient id 2015004).

The optimal concentrations of the expressed *P. falciparum* antigens were established by serial dilutions in a series of FluoroSpot assays and defined as the dilution where the number of counted spots plateaued without increasing background noise. The concentration was set to 1:50 dilution for all antigens except AMA-1, which was used at a 1:500 dilution (**Figure 2A**). When multiplexing the antigens, MBC reactivity against the four *P. falciparum* antigens MSP-1_19_, MSP-2, MSP-3 and AMA-1 could be simultaneously detected in the same well as exemplified using PBMCs from one of the donors in a sample collected one month after treatment (**Figure 2B**). Similar frequencies of counted SFU and median RSV of counted spots were detected when the FluoroSpot assay was performed with each antigen alone (singleplex) or when all four antigens were included (multiplex) (**Figure 3A**). Moreover, SFU and median RSV were similar between tests when the assay was performed on the same samples on two separate occasions (**Figure 3B**).

**Figure 4:**
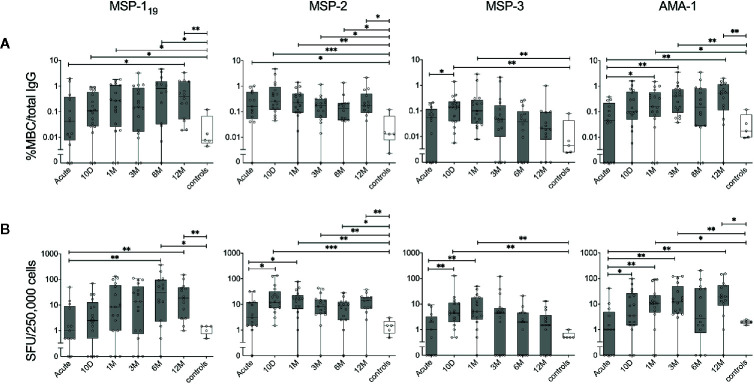
Kinetics of the parasite-specific memory B-cell (MBC) reactivity in individuals treated for acute *P. falciparum* malaria. B-cell reactivity against peptide-tagged MSP-1_19_, MSP-2 (3D7 combined with FC27), MSP-3 and AMA-1 in individuals treated for acute malaria (grey) as well as healthy unexposed controls (white) was measured by FluoroSpot at different time points and presented as **(A)** percentage of antigen-specific MBCs per total number of total IgG spots, or **(B)** frequency of spot-forming units (SFU) per 250,000 PBMCs. Differences between timepoints were evaluated by Mann-Whitney test. *P < 0.05, **P < 0.01, ***P < 0.01.

MBC responses were measured against MSP-1_19_, MSP-2, MSP-3 and AMA-1 using the FluoroSpot assay in 20 travellers diagnosed with acute *P. falciparum* malaria sampled at 4-6 timepoints per donor over one year. All had fever and other symptoms indicative of malaria with a symptom duration of median 5 days (range 0-9 days) and two patients had severe malaria (**Supplementary Table 1**). All but one individual (a first-time infected traveller), had MBCs against at least one of the antigens on at least one time point during the study period (**Figures 4** and **9**). Reactivities against control antigens tagged with same peptide tags as the *P. falciparum* antigens were low during the whole study period and did not differ from the reactivity of healthy malaria unexposed controls (**Supplementary Figure 2**). In total 24 (20%) individual time points were not included due to missing samples (n=14, 11.6%) or disqualified assays (n=10, 8.3%) i.e. when <1,000 total IgG SFU per 250,000 cells were obtained.

### Kinetics of MBC Responses in Individuals Treated for a *P. falciparum* Malaria Infection

The kinetics of the MBC responses were assessed during the one year follow up. Despite a high degree of variability between individuals, the median proportion of MBCs against each of the antigens were comparable to healthy controls at the acute time point and then increased at later time points for most antigens (**Figure 4**). The exception to this was MBC responses to MSP-2 that were higher in the infected individuals compared to healthy controls at all time-points. The increase in the MBC response at the later time-points was detected both when assessed as proportion antigen-specific MBC per total IgG producing MBC (%MBC/total IgG) (**Figure 4A**), and as MBC per 250,000 cells (i.e the number of cells added per well) (**Figure 4B**), respectively. For some antigens, the MBC response increased significantly already at day 10 whereas after 12 months, only the MBC response to MSP1_19_ and AMA-1 were significantly elevated compared to the acute time point as well as MBC response of the healthy controls (**Figures 4A, B** **)**. Furthermore, the kinetics of the MBC response were heterogenous between antigens. For instance, while the median proportion of MBCs to MSP-1_19_ was approximately 50 times higher than healthy controls after 12 months, MBC responses to MSP-3 were comparable to that of healthy controls after 12 months (**Figure 4**). Similarly, at the acute time point, MBC responses against MSP-2 were approximately 12 times higher compared to healthy controls while responses to AMA-1 were comparable to healthy control (**Figure 4**).

**Figure 5:**
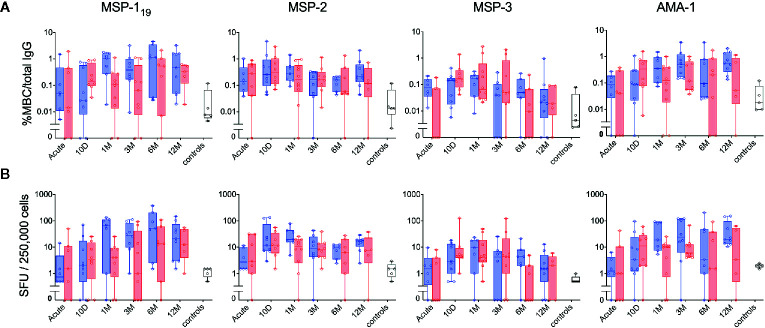
Kinetics of the parasite-specific memory B-cell (MBC) and antibody reactivity in individuals with different history of exposure. B-cell reactivity against peptide-tagged MSP-1_19_, MSP-2 (3D7 combined with FC27), MSP-3 and AMA-1 in primary infected (blue) and previously exposed (red) individuals as well as healthy unexposed controls (white) was measured by FluoroSpot at different time points and presented as **(A)** percentage of antigen-specific MBCs per total number of total IgG spots, or **(B)** frequency of spot-forming units (SFU) per 250,000 PBMCs. Differences between timepoints were evaluated by Mann-Whitney test.

When the MBC responses were compared between primary infected and previously exposed individuals for each of the four antigens, no significant differences were observed at any timepoint, irrespective of whether the frequencies were expressed as %MBC/total IgG (**Figure 5A**) or as SFU (**Figure 5B**). Similarly, when the kinetics of MBC responses between groups were compared using a linear mixed effects model, no differences were measured (**Supplementary Table 2**). The kinetics of *P. falciparum* antigen-specific MBC responses at the individual level can be also seen in **Supplementary Figure 3**. Regarding severity of disease, we did not see any indications that the two patients (patient ID 2014005 and 2015003) with severe malaria differed in their MBC or antibody responses compared to the other patients.

When time since residency in an endemic area was correlated to MBC responses for previously exposed individuals, no significant association were detected against any antigen with the exception of MSP-1 that showed a positive correlation with time since residency in an endemic area (Spearman correlation coefficient r_s_ 0.429 p=0.003, r_s_ 0.211 p=0.16, r_s_ −0.178 p=0.237 and r_s_ 0.185 p= 0.217 for MSP-1_19_, MSP-2, MSP-3 and AMA-1 respectively).

### Relative Spot Volume

**Figure 6:**
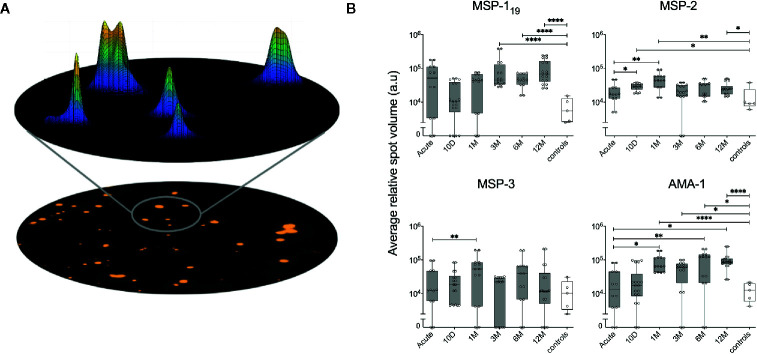
Assessment of relative spot volume (RSV) and the kinetics of RSV against *P. falciparum* antigens in individuals treated for acute malaria. **(A)** Schematic model of the acquisition of spot volume. The RSV of single spots is determined by a mathematical diffusion model performed by the software algorithms. Based on the calculated shape of spots, the relative spot volume is then calculated as the area under curve. **(B)** Average RSV of counted spots against *P. falciparum* antigens in individuals treated for acute malaria (grey) as well as healthy unexposed controls (white) at different time points. Differences between timepoints were evaluated by Mann-Whitney test. *P < 0.05, **P < 0.01, ****P < 0.0001.

The Mabtech IRIS™ FluoroSpot reader software Apex™ provides information about the volume and intensity of each of the spots counted by the reader, presented as relative spot volume (RSV) (25) (**Figure 6A**). When the kinetics of average RSV from spots against MSP-1_19_, MSP-2, MSP-3 and AMA-1 were plotted (**Figure 6B**), the average RSV showed similarities to the kinetics seen for %MBC/total IgG and SFU/250,000 (**Figures 4A, B**). For MSP-1_19_ there was a significant increase of average RSV compared to healthy controls at the later time points. For MSP-2, the average RSV was significantly increased between the acute time point and the earlier time points. There was also a significant increase of average RSV against MSP-2 between the healthy controls and at the 12-month follow up as well as earlier time points. For MSP-3, there was a significant increase in average RSV between the acute time point and the 10-day follow-up. Finally, the average RSV of MBC responses against AMA-1 was significantly increased between the acute time point and at the later time points; and average RSV against AMA-1 was also significantly higher compared with the healthy controls at all time points except at the acute and 10-day follow up (**Figure 6B**).

**Figure 7:**
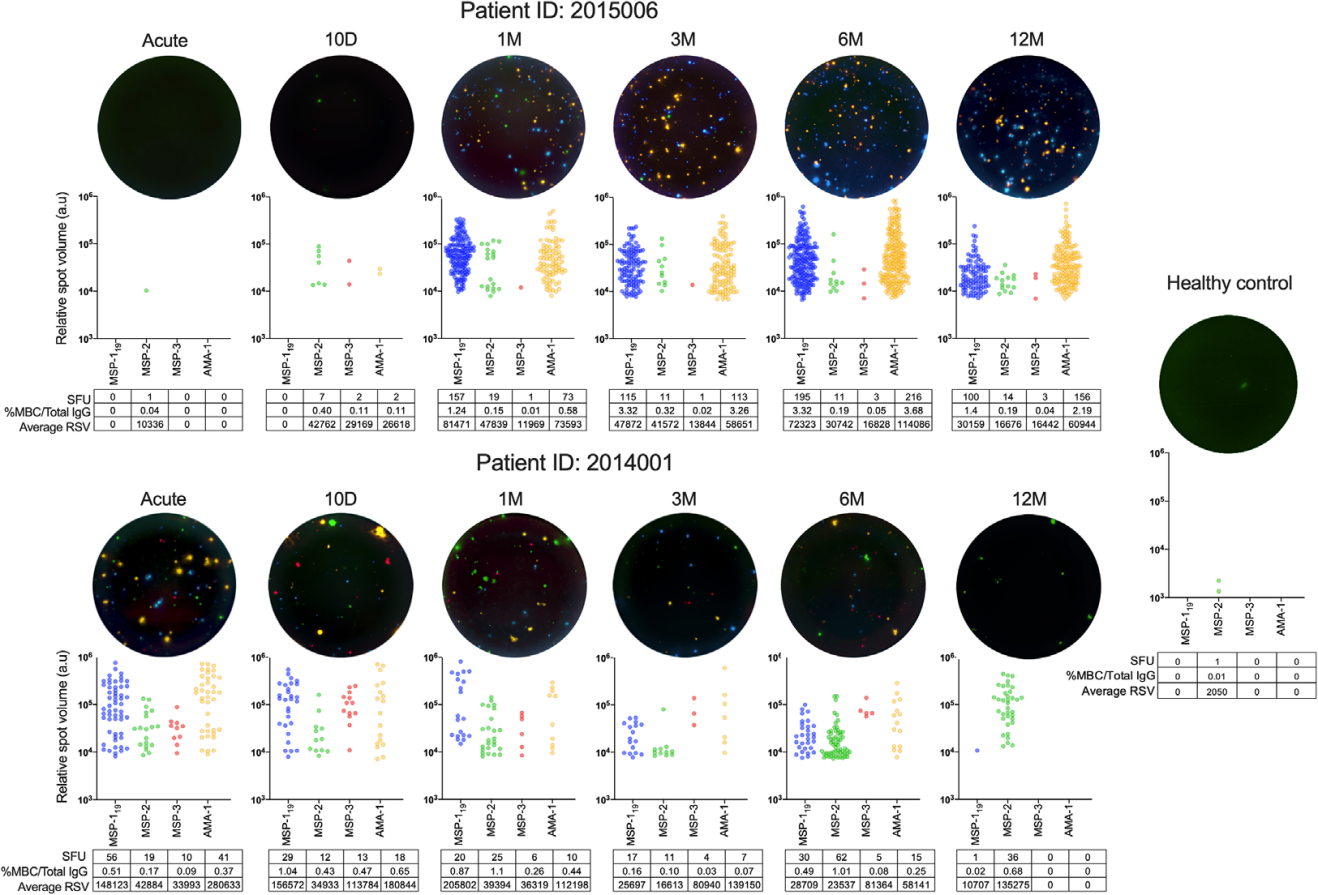
B-cell responses and relative spot volume (RSV) of individual spots against *P. falciparum* antigens from two individuals at different time points over one year follow up and healthy controls measured by FluoroSpots and a healthy control as measured by FluoroSpot. Single wells from two donors at different timepoints as well as a healthy control presented as computerized overlay of filter image, RSV value of individual spots counted for all four antigens, and the frequency of spot forming units (SFU) per 250,000 PBMCs, percentage of antigen-specific memory B cells per total IgG, and average RSV of all counted spots.

When average RSV and %MBC/total IgG were compared, a positive correlation for MSP-1_19_, MSP-3 and AMA-1 could be measured (**Supplementary Figure 4A**). Furthermore, The kinetics of frequency of spots and RSV within two individuals (ID2015006 and ID 2014001) are illustrated in **Figure 7**, even though the selected individuals are examples and do not represent the kinetics of all individuals in their respective subgroup. 

### Kinetics of Plasma Antibody Responses

**Figure 8:**
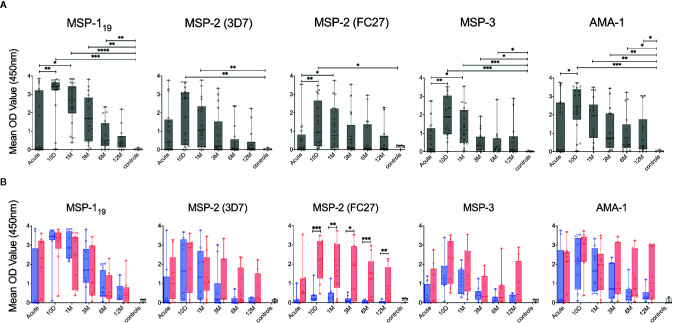
Kinetics of *P. falciparum* antigen-specific antibodies in individuals treated for acute malaria. **(A)** Antibody levels in plasma against *P. falciparum* antigens in all individuals treated for acute malaria at different time points as well as healthy malaria unexposed controls (white) as measured by ELISA using TWIN-Strep^®^-tagged antigens. **(B)** Antibody levels in plasma against *P. falciparum* antigens in primary infected (blue) and previously exposed (red) individuals as well as healthy unexposed controls (white) as measured by ELISA using TWIN-Strep^®^-tagged antigens. Differences between timepoints were evaluated by Mann-Whitney test. *P < 0.05, **P < 0.01, ***P < 0.001, ****P < 0.0001.

Antibody responses in plasma against MSP-1_19_, MSP-2 (3D7), MSP-2 (FC27), MSP-3 and AMA-1 were measured by peptide tag ELISA with TWIN-Strep^®^ tagged variants of the antigens (**Figure 8A**). For all antigens tested, antibody levels peaked around 10 days after diagnosis and then declined over the study period (**Figure 8A**), although this decline was more rapid for the primary infected group (**Figure 8B**). When antibody levels were compared between primary infected and previously exposed individuals in a linear mixed effects model adjusted for time, the previously exposed group had significantly higher levels of antibodies against MSP-2 (FC27), MSP-3 and AMA-1 compared to the primary infected group (**Supplementary Table 2**) as previously reported in this cohort (5). When antibody levels towards the different antigens were compared at specific time points, previously exposed individual had significantly higher levels of antibodies against MSP-2 (FC27) at several time points compared to primary infected individuals (**Figure 8B**).

Limited correlation was observed between plasma antibody levels measured by ELISA and MBC responses measured by FluoroSpot (**Supplementary Figure 4B**). An exception to this was antibody levels against MSP-2 (3D7) and MSP-3 for which there was a positive correlation with %MBC/total IgG (**Supplementary Figure 4B**).

### Breadth of MBC and Antibody Responses Over Time

**Figure 9:**
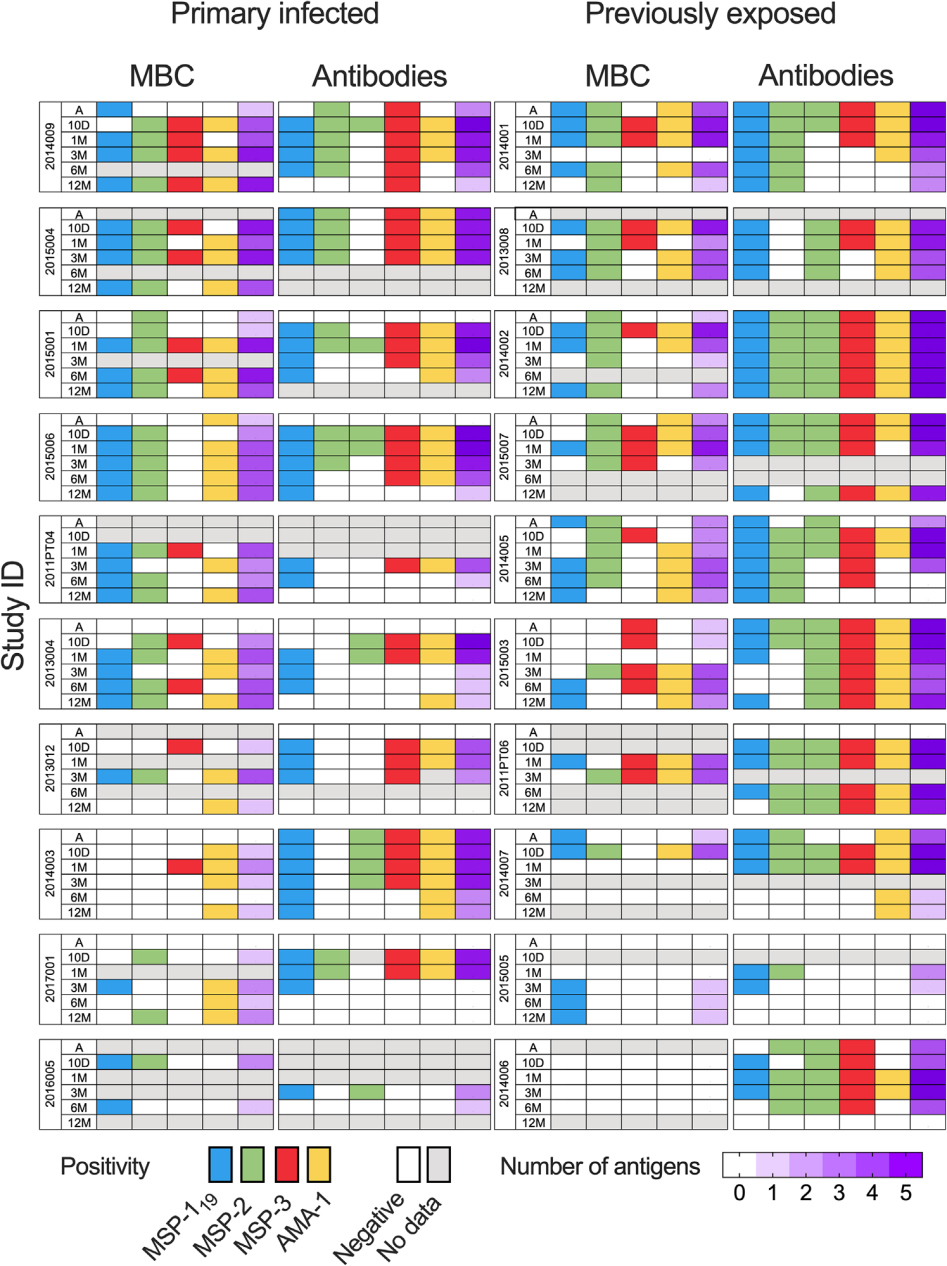
Breadth of memory B-cell (MBC) and antibody responses against *P. falciparum* antigen in individuals treated for acute malaria. Breadth was defined by the number of antigens an individual had responses above threshold for positivity. Positivity and breadth in study participants at different timepoints. Positivity, defined as a response above mean response of healthy unexposed controls +2 SD, are presented as colored cells. Blue cells represents positivity against MSP-1_19_, green: MSP-2, red: MSP-3 and yellow: AMA-1. Cells with white indicate negative and grey loss of sample data due to missing data. Breadth is presented by increasing purple gradient with increasing number of antigens positive against.

The breadth of the response was defined as the number of antigens against which an individual had reactivity above MBC or seropositivity threshold (i.e. mean response of healthy unexposed controls +2 SD), respectively. The number of individuals that were considered MBC positive against an antigen, on at least one time point during the study period, was highest for MSP-1_19_ with positivity in 18 (90%) individuals, followed by AMA-1 and MSP-2 in 17 (85%), and MSP-3 in 14 (70%) of the individuals (**Figure 9**). Including all timepoints, 13 (65%) individuals were positive against all four antigens (**Figure 9**).

The breadth of MBC responses was lowest at the acute time point, with responses to a median of 1 (range 0–3) antigen, and then increased at the later time points. The breadth of MBC responses was highest at the 1 month-follow up (median 3 range 0–4) and tended to decline thereafter (**Figure 9**). No significant differences were found when comparing the breadth of MBC responses between primary infected and previously exposed individuals over time (**Supplementary Table 2**).

The breadth of antibody responses in plasma was highest at the 10-day and 1-month follow-up but then declined at the later timepoints (**Figure 9**). When primary infected and previously exposed individuals were compared, breadth of circulating antibodies was significantly higher in the previously exposed compared to the primary infected group throughout the study period (**Supplementary Table 2**).

## Discussion

Here, we developed a reversed B-cell FluoroSpot assay that simultaneously detects antigen-specific MBCs to the four different merozoite antigens (MSP-1_19_, MSP-2, MSP-3, and AMA-1) in PBMCs from individuals followed prospectively after an acute episode of *P. falciparum* malaria. We demonstrated the quality and functionality of the multiplex assay to study antigen-specific MBC responses ex vivo. By analysing the MBC response at several time points over one year following treatment in a malaria free setting, we could study the kinetics of the response during and after the acute infection without the risk of re-infection. We show heterogeneity in responses to the four included antigens within and between individuals, and that both primary infected and previously exposed individuals were able to acquire and maintain merozoite antigen-specific MBCs one year after natural acute infection.

The role of MBCs detected in peripheral blood has not been clearly demonstrated in human malaria. The reasons may be partly technical, owing to difficulties in measuring very low frequencies of antigen-specific MBCs in circulation. The challenge is even more complicated by the multiplicity and diversity of *P. falciparum* antigens to be studied, as well as limitations of getting enough cells to study MBC responses to several antigens. Clearly, more sensitive methods than multiple ELISpot assays are needed for increased capacity and efficiency. The novel FluoroSpot assay showed high sensitivity and specificity to the four include *P. falciparum* merozoite antigens and the possibility to multiplex in a simultaneous assay.

Previous studies using ELISpot from Mali and Thailand have found that in areas of seasonal low transmission, MBCs responses against merozoite antigens can be maintained for an extended period of time in the absence of reinfection (13, 14). This was also shown in our previous study in traveller treated for malaria in Sweden, where approximately 80% of the individuals had detectable MBCs against at least one antigen and 20% had reactivity against all three antigens (MSP-1_42_, MSP-3 and AMA-1), as determined by ELISpot several years after infection (11). In the present study, we had the opportunity to study the kinetics of response to four merozoite antigens over one year without re-exposure. The primary infected and the previously exposed individuals displayed similar MBC levels, although the number of individuals in each group was limited. This was notable since the previously exposed individuals presented with more rapid and higher magnitudes of circulating antibodies in plasma, possibly reflecting an activated memory response of MBCs from previous exposure. Future studies should involve larger number of individuals, in order to confirm this. Also, since history of previous infections before the current acute malaria episode were self-reported, we could not define the exact time since last previous infection or the presence of asymptomatic infections. We also lacked information regarding the potential previous exposure in primary infected individuals and whether they had resided repeatedly in high transmission areas even with chemoprophylaxis. These factors could possibly have influenced the levels of *P. falciparum*-specific MBCs in circulation in these individuals.

All, but one individual (a primary infected traveller) followed after acute *P. falciparum* malaria were MBC positive against at least one merozoite antigen during the one-year observation period. A subset (35%) of individuals had MBC responses against all four merozoite antigens at a single time point. Heterogeneity of the MBC responses, also within individuals, may be explained by low frequencies of MBCs in circulation, falling under the detection limit of the assay in individual samples. Importantly, most individuals (65%) were positive against all antigens at some time during the one year follow up, demonstrating the detection sensitivity of our assay as well as the advantage of following patients over time compared to investigating just a single time point.

The MBC positivity was highest for MSP-1 and AMA-1, moderate for MSP-2, while lower against the MSP-3 antigen. While MBC responses to MSP-1 and AMA-1 showed tendencies of increasing over time, magnitude of MBC responses to MSP-2 and MSP-3 seemed less pronounced at the later timepoints compared to the earlier timepoints. In support of this, other studies have also demonstrated detectable MBC responses against MSP-1_19_ and AMA-1 that were maintained for an extended period of time (11, 13), suggesting that the immunogenicity of *P. falciparum* merozoite antigens and to what extent they induce long-lived MBC in circulation differs.

Considering the high number of antigens expressed during the parasite life cycle, the proportion of antigen-specific MBCs (up to 4.6% of total IgG MBC for MSP-1_19_) observed after a primary infection was remarkably high in some individuals. A potential reason for this is that MSP-1 is the most abundantly expressed protein on the parasite surface (26) and has been shown to be expressed also at earlier phases of the parasite life cycle (27), hence, allowing for longer stimulation of the B-cell response during the infection. MSP-1 is therefor often used as a serological marker of past exposure (5, 28). In addition, MSP-1_19_ is highly conserved (29) compared to MSP-2 and MSP-3, which have a higher level of genetic polymorphisms (30, 31). Hence, the antigens included in the assay may differ in the polymorphic regions from the specific antigen variants to which the patients were exposed during their infection, which could explain the low antibody reactivity of primary infected individuals against MSP-2 (FC27), hence also potentially affecting the sensitivity of the assay for polymorphic antigens. Nevertheless, when combining the two major allelic variants of MSP-2, MBC responses against this highly polymorphic merozoite antigen could be detected also in primary infected individuals.

Loss of antigen-specific MBCs over one year could possibly be explained by a reduction in numbers to below detection level, as well as relocation of MBCs into secondary lymphoid organs, such as the spleen or lymph nodes, where during a healthy state, antigen-specific MBCs have been reported to reside in higher frequencies compared to circulating MBCs (32). Activation of these splenic MBCs could then lead to the differentiation of MBCs into plasma blasts boosting level of antibodies in circulation, reflected by the increased magnitude of antibody responses observed for previously exposed individuals in this cohort (5). This gradual loss of detectable MBCs in peripheral blood is in line with our previous study performed several years after acute infection that demonstrated a lower proportion of individuals positive for MBC responses against merozoite antigens compared to this study (11).

The reversed FluoroSpot was here adapted to four merozoite surface antigens for which we have investigated serological responses to before (5). The use of peptide-tagged antigens as well as anti-tag detection antibodies facilitates the possibility to adapt the assay for the analysis of other antigens of interest. Although other methods, such as flow cytometry can be used to study antigen-specific MBCs (33), the FluoroSpot assay is more robust, less laborious and allow high throughput analysis of MBC responses to a higher extent compared to flow cytometry. Moreover, as we have previously shown, the B-cell FluoroSpot assay can be used to study cross-reactivity displayed by single B cells (34). Indeed, the B-cell FluoroSpot assay used herein could also be used to explore cross-reactivity against highly polymorphic antigens, an aspect that is further investigated within this cohort.

Moreover, novel modalities of the FluoroSpot reader allow further exploration of the B-cell response by measuring size and intensity, i.e. the RSV value, of the counted spots (29). Our results indicate a gradual increase in average RSV against MSP-1_19_ and AMA-1 with time. This increase could potentially reflect affinity maturation of the B-cell compartment. In accordance with this, we have previously shown an increase in median RSV after vaccination against hepatitis B virus in previously unvaccinated volunteers (21). Furthermore, average RSV against all antigens with the exception of MSP-2, showed a moderate correlation with %MBC/total IgG. Even though the biology of the correlation remains unknown, the possibility of extracting detailed information regarding single spots provides an additional dimension to the FluoroSpot analysis of spot frequency, and further studies will have to explore to what extent RSV values reflect the quality of the immune response.

In conclusion, the multiplex reversed B-cell FluoroSpot assay that we developed can simultaneously detect MBCs producing antibodies against four different *P. falciparum* merozoite surface antigens. Using this assay, we show that it is possible to detect parasite-specific MBCs up to one year after previous infection and that the magnitude and breadth of the MBC response does not markedly differ between primary and previously exposed individuals. Our results suggest that immunological memory against merozoite antigens is established in travellers after a first single *P. falciparum* infection, although this memory is not sufficient for protection against new infections. These findings provide new insights toward the acquisition and maintenance of MBCs after a natural infection of *P. falciparum*. We believe that this new multiplex FluoroSpot assay will be a highly valuable tool for studies that aim to further explore immunological memory to the variety of antigens of the complex malaria parasite.

## Data Availability Statement

The data sets used/or analyzed in the current study are available from the corresponding authors upon reasonable request.

## Ethics Statement

The studies involving human participants were reviewed and approved by Central Ethical Review Board in Stockholm Dnr 2006/893-224 31/4, 2013/550-32, 2018/2354-32. The patients/participants provided their written informed consent to participate in this study.

## Author Contributions

PJ designed and performed the study, analyzed and interpreted the data, and prepared the manuscript. CSu assisted in the design of the study, assisted with interpretation of data, supervised the study, and revised the manuscript. VY assisted in sample collection, interpretation of the data, and data analysis. LW assisted with statistical analysis of data. MA assisted in sample collection and interpretation of the data. KS and CSt assisted in sample collection and patient handling. CSm assisted with technical expertise and interpretation of data. FN assisted with interpretation of data. AF designed the study, supervised the study implementation, analyzed the data, and revised the manuscript. All authors contributed to the article and approved the submitted version.

## Funding

This work was funded by The Swedish Foundation for Strategic Research (ID14-0070), project grants from the Swedish Research Council (VR MH 201502977 and 201802688) and the Stockholm County Council (ALF 20150135 and 20180409).

## Conflict of Interest

PJ, CSm, and NA are employed by Mabtech AB, Sweden. Several of the reagents and the FluoroSpot reader system used in this study are produced by Mabtech. Mabtech has no influence on the content of this study.

The remaining authors declare that the research was conducted in the absence of any commercial or financial relationships that could be construed as a potential conflict of interest.
