# Supplementary material for: Memory B-Cell Responses Against Merozoite Antigens After Acute Plasmodium falciparum Malaria, Assessed Over One Year Using a Novel Multiplexed FluoroSpot Assay
Source: Front Immunol. 2021 Feb 12;11:619398. doi: 10.3389/fimmu.2020.619398 (PMC7928423; doi:10.3389/fimmu.2020.619398)
Supplement: Supplementary file 1 [file Table_1.docx]

**Supplementary table 1. Study population**

|  | **Primary infected** | **Previously exposed** | **All** |  |
| --- | --- | --- | --- | --- |
| Number of participants | 10 | 10 | 20 |  |
| Female | 3 | 2 | 5 |  |
| Age. years median (range) | 38 (25-48) | 51 (32-68) | 45 (25-68) |  |
| Time lived in endemic area. years median (range) | 0 (0-2) | 24.5 (18-39) | 18 (0-39) |  |
| Time since residency in endemic area. years median (range) | - | 18.5 (2-32) | 9 (0-32) |  |
| Time from symptom onset to diagnosis. days median (range) | 5 (0-7) | 4 (1-9) | 5 (0-9) |  |
| Max infected red blood cells during treatment %median (range) | 0.8 (0.01-5.6) | 0.2 (0.01-17) | 0.5 (0.01-17) |  |
| Severe malaria^1^ | 0 | 2 | 2 |  |
| Treatment failure^2^ | 4 | 0 | 4 |  |
|  |  |  |  |  |
|  |  |  |  |  |
|  | | |  |  |
|  | |  |  |  |

*^1^Severe malaria according to the criteria set by the WHO 2014 (One patient suffered acidosis another low blood preasure)*

*^2^Patients who presented with recrudecent* P. falciparum *parasitemia 7-35 days after treatment*
