# Supplementary material for: Memory B-Cell Responses Against Merozoite Antigens After Acute Plasmodium falciparum Malaria, Assessed Over One Year Using a Novel Multiplexed FluoroSpot Assay
Source: Front Immunol. 2021 Feb 12;11:619398. doi: 10.3389/fimmu.2020.619398 (PMC7928423; doi:10.3389/fimmu.2020.619398)
Supplement: Supplementary file 2 [file Table_2.docx]

**Supplementary table 2. Comparison of responses between primary and previously exposed individuals**

A mixed-effects linear regression model adjusted for time of sample together with subject specific random intercept and time slope was used to compare differences between groups "primary infected" and "previously exposed” regarding memory B-cell responses and antibody levels across all timepoints.

*^1^ Primary infected patients with recrudescent* P.falciparum *malaria were compared with individuals in the same group.*
