# Supplementary figures and images for: Memory B-Cell Responses Against Merozoite Antigens After Acute Plasmodium falciparum Malaria, Assessed Over One Year Using a Novel Multiplexed FluoroSpot Assay
Source: Front Immunol. 2021 Feb 12;11:619398. doi: 10.3389/fimmu.2020.619398 (PMC7928423; doi:10.3389/fimmu.2020.619398)

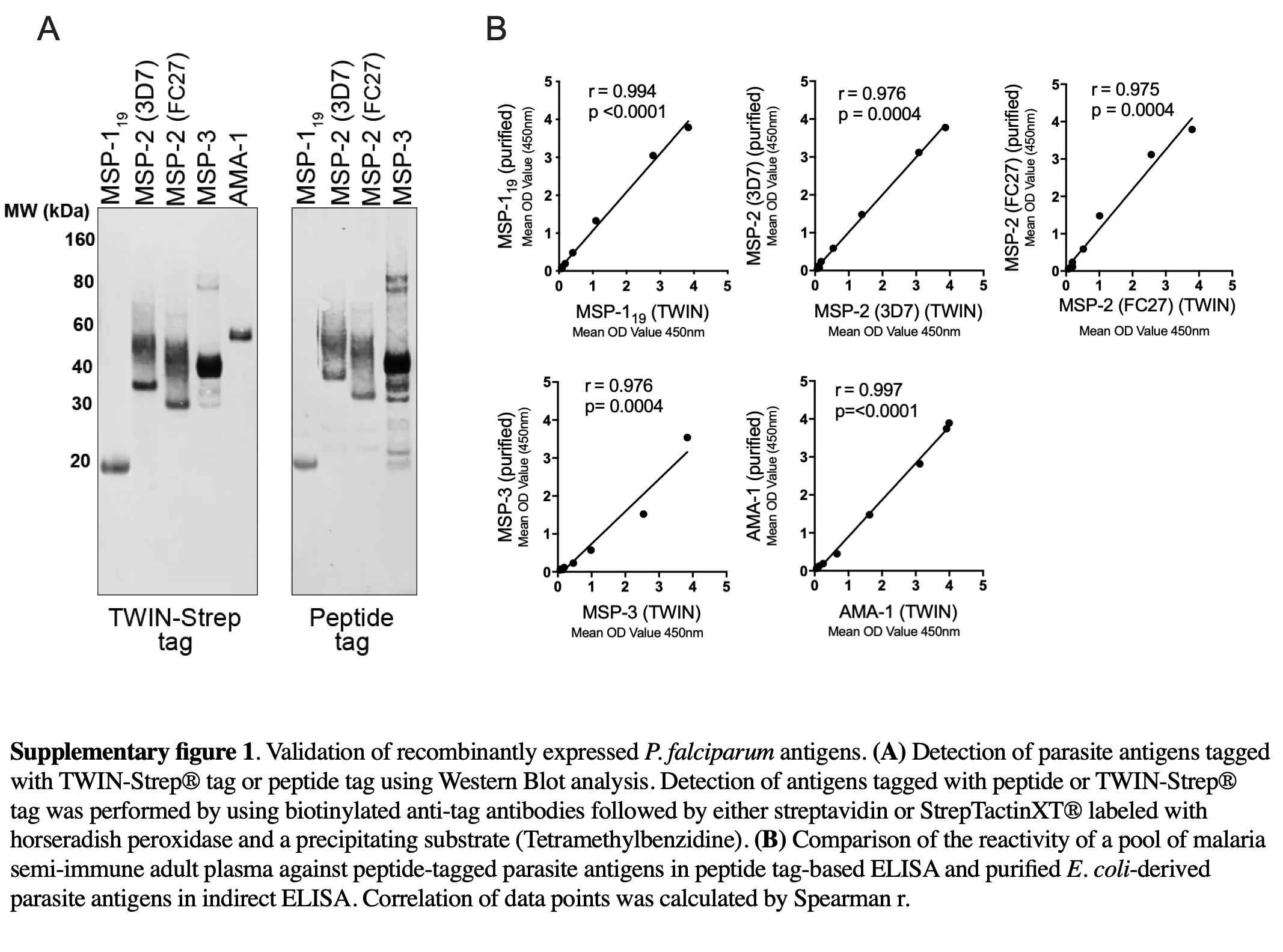

Supplement: Supplementary file 3 [file Image_1.tiff]

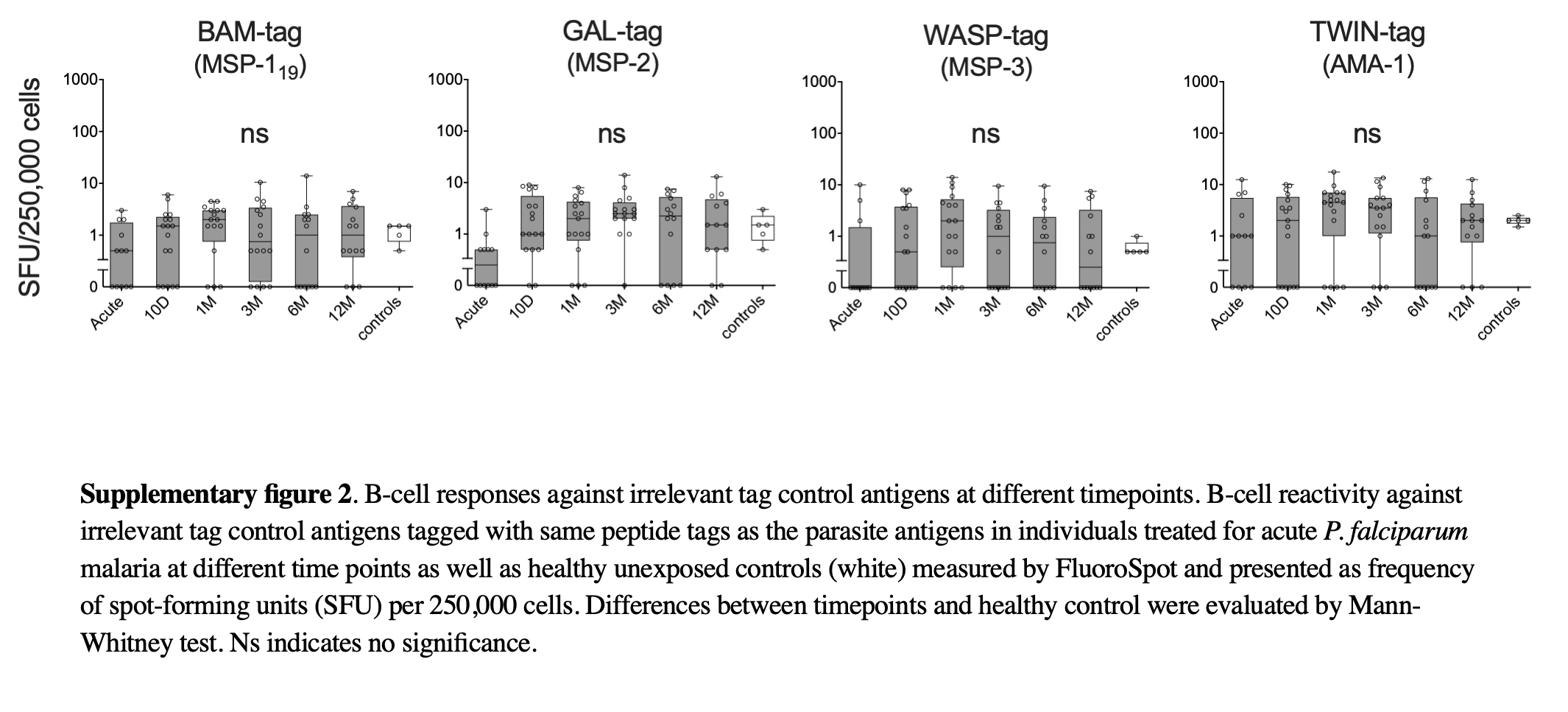

Supplement: Supplementary file 4 [file Image_2.tiff]

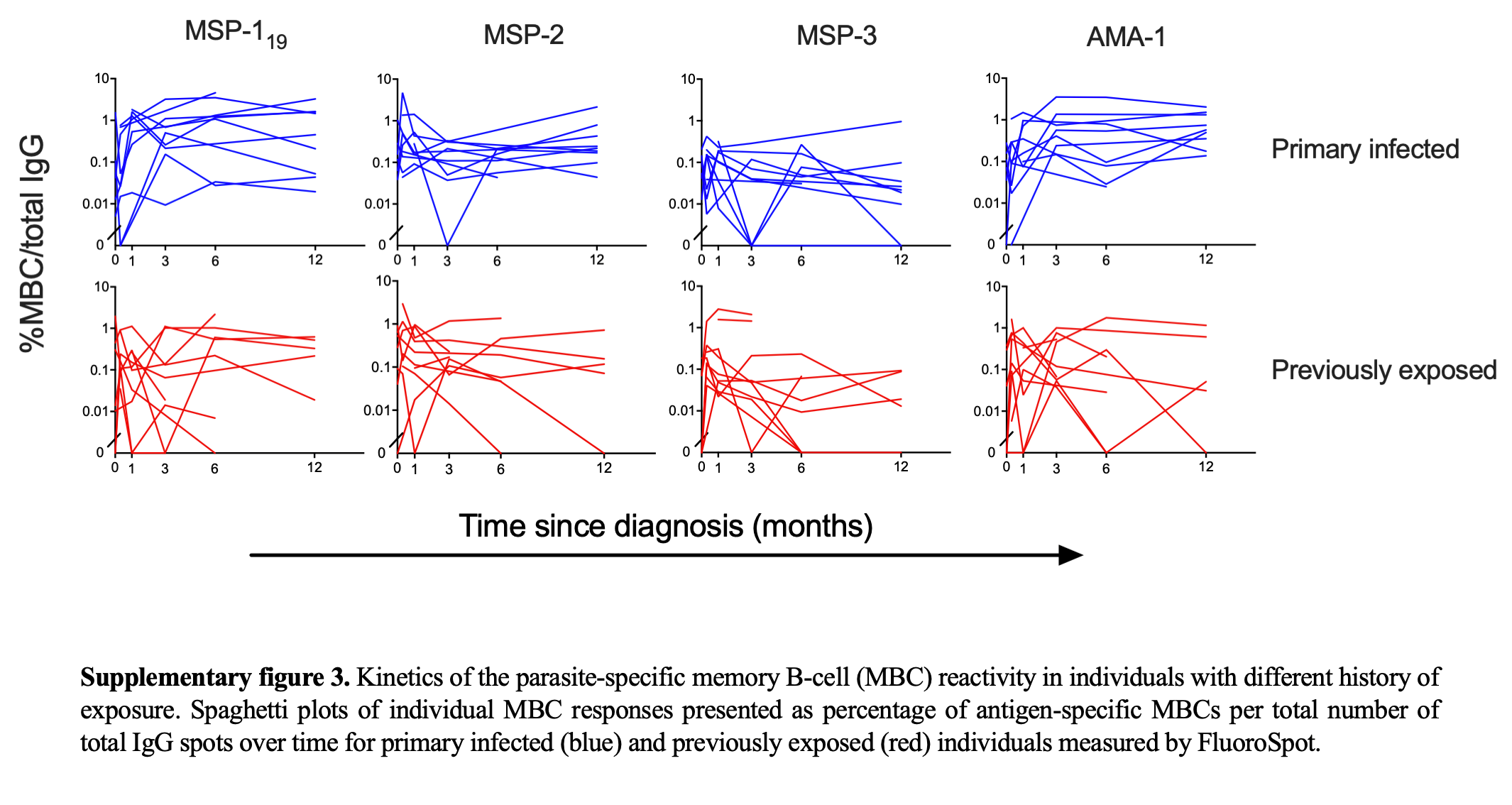

Supplement: Supplementary file 5 [file Image_3.tiff]

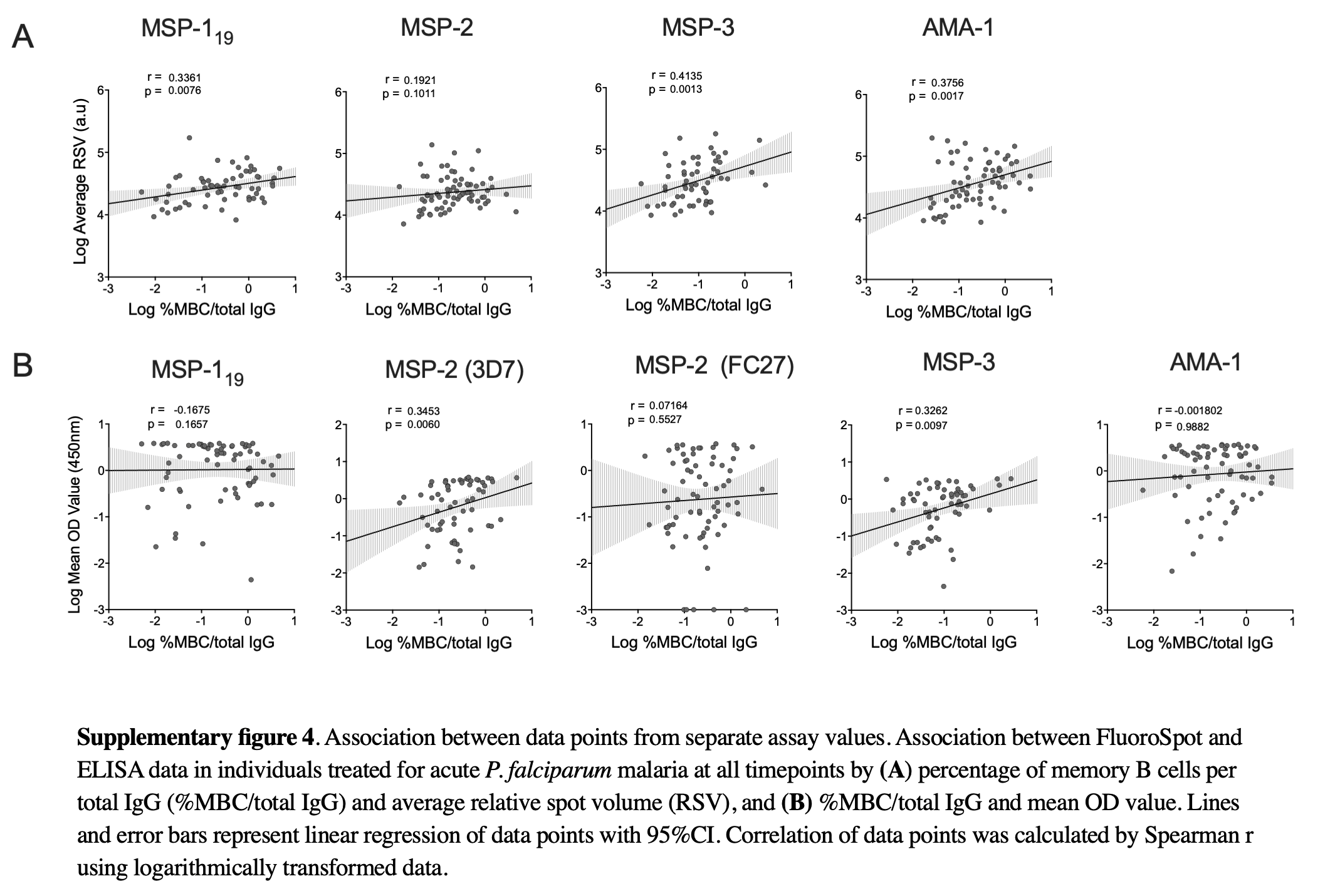

Supplement: Supplementary file 6 [file Image_4.tiff]
